# Epigenetic landscapes suggest that genetic risk for intracranial aneurysm operates on the endothelium

**DOI:** 10.1186/s12920-019-0591-7

**Published:** 2019-10-30

**Authors:** Kerry E. Poppenberg, Kaiyu Jiang, Michael K. Tso, Kenneth V. Snyder, Adnan H. Siddiqui, John Kolega, James N. Jarvis, Hui Meng, Vincent M. Tutino

**Affiliations:** 1 Clinical and Translational Research Center, Canon Stroke and Vascular Research Center, 875 Ellicott Street, 14203 Buffalo, NY USA; 2 Department of Biomedical Engineering, University at Buffalo, Buffalo, NY USA; 3 Genetics, Genomics, and Bioinformatics Program, Jacobs School of Medicine & Biomedical Sciences, University at Buffalo, Buffalo, NY USA; 4 Department of Neurosurgery, Jacobs School of Medicine & Biomedical Sciences, University at Buffalo, Buffalo, NY USA; 5 Department of Radiology, Jacobs School of Medicine & Biomedical Sciences, University at Buffalo, Buffalo, NY USA; 6 Department of Pathology and Anatomical Sciences, Jacobs School of Medicine & Biomedical Sciences, University at Buffalo, Buffalo, NY USA; 7 Department of Pediatrics, Jacobs School of Medicine & Biomedical Sciences, University at Buffalo, Buffalo, NY USA; 8 Department of Mechanical & Aerospace Engineering, University at Buffalo, Buffalo, NY USA

**Keywords:** Cell type, Cerebral aneurysm, Epigenetics, Genetics, Risk

## Abstract

**Background:**

Genetics play an important role in intracranial aneurysm (IA) pathophysiology. Genome-wide association studies have identified several single nucleotide polymorphisms (SNPs) that are linked to IA but how they affect disease pathobiology remains poorly understood. We used Encyclopedia of DNA Elements (ENCODE) data to investigate the epigenetic landscapes surrounding genetic risk loci to determine if IA-associated SNPs affect functional elements that regulate gene expression and if those SNPs are most likely to impact a specific type of cells.

**Methods:**

We mapped 16 highly significant IA-associated SNPs to linkage disequilibrium (LD) blocks within the human genome. Within these regions, we examined the presence of H3K4me1 and H3K27ac histone marks and CCCTC-binding factor (CTCF) and transcription-factor binding sites using chromatin immunoprecipitation-sequencing (ChIP-Seq) data. This analysis was conducted in several cell types relevant to endothelial (human umbilical vein endothelial cells [HUVECs]) and inflammatory (monocytes, neutrophils, and peripheral blood mononuclear cells [PBMCs]) biology. Gene ontology analysis was performed on genes within extended IA-risk regions to understand which biological processes could be affected by IA-risk SNPs. We also evaluated recently published data that showed differential methylation and differential ribonucleic acid (RNA) expression in IA to investigate the correlation between differentially regulated elements and the IA-risk LD blocks.

**Results:**

The IA-associated LD blocks were statistically significantly enriched for H3K4me1 and/or H3K27ac marks (markers of enhancer function) in endothelial cells but not in immune cells. The IA-associated LD blocks also contained more binding sites for CTCF in endothelial cells than monocytes, although not statistically significant. Differentially methylated regions of DNA identified in IA tissue were also present in several IA-risk LD blocks, suggesting SNPs could affect this epigenetic machinery. Gene ontology analysis supports that genes affected by IA-risk SNPs are associated with extracellular matrix reorganization and endopeptidase activity.

**Conclusion:**

These findings suggest that known genetic alterations linked to IA risk act on endothelial cell function. These alterations do not correlate with IA-associated gene expression signatures of circulating blood cells, which suggests that such signatures are a secondary response reflecting the presence of IA rather than indicating risk for IA.

## Background

Intracranial aneurysms (IAs) are characterized by aberrant gene expression consistent with inflammatory cell infiltration and immune cell-regulated vascular degeneration [1–5]. The presence of abnormal transcriptional signatures in the peripheral blood of patients with IAs has also been observed in several recently published reports [6–10]. In a case-controlled study, we performed transcriptome profiling of circulating neutrophils in patients with and without IAs [6]. Differential expression analysis revealed statistically significant differentially expressed transcripts that were related to increased peripheral neutrophil activation. These findings led us to question whether the aberrant peripheral blood gene expression signatures are the result of an interaction between these circulating neutrophils and the diseased aneurysmal tissue or whether the formation of the aneurysm is due to dysregulated immune or inflammatory processes that might be attributed partly to genetics.

Genetics play an important role in IA pathophysiology. Patients with certain hereditary diseases (i.e., Ehlers-Danlos syndrome, Marfan syndrome, Neurofibromatosis-1) are known to have higher rates of IAs (10–20%) and aneurysm rupture (8–25%) compared to the general population [11]. Many mutations associated with these conditions (e.g., collagen [*COL1A*1] in patients with Ehlers-Danlos syndrome) affect the structural integrity of the vasculature or the ability of the vessel walls to maintain homeostasis [12, 13]. A family history of IA is also associated with higher IA prevalence (10%) and rupture rates (4%) [14], suggesting other heritable genetic factors contribute to disease susceptibility. Genome-wide association studies (GWAS) using large cohorts from principally Dutch, Finnish, and Japanese populations have identified many single nucleotide polymorphisms (SNPs) that occur more commonly in individuals with IAs [15–23]. In these studies, significant associations were reported at 2q32.1 (*PLCL1*) [16], 8q11.23–q12.1 (*SOX17*) [16], 9p21.3 (*CDKN2A*-*CDKN2B*) [16], 18q11.2 (*RBBP8*) [23], 13q13.1 (*STARD13*) [23], and 10q24.32.12 [23]. The most frequently replicated locus has been 9p21.3 at the noncoding RNA, *CDKN2B*–*AS1*, which is in the *CDKN2B*-*CDKN2A* gene cluster, and has been shown to be a significant genetic susceptibility locus for cardiovascular diseases [24]. In a meta-analysis, Alg et al. [25] investigated 66 case-controlled studies that included 32,887 IA patients and 83,683 control subjects and identified 19 SNPs that were significantly associated with IAs, the most replicated of which were at 9p21.3, 8q11, and 4q31.23. Like those in association with other complex diseases or traits [26–29], several IA-risk loci have been found in noncoding regions of the genome, suggesting that genetic risk may operate on functional regulatory elements that influence gene expression, rather than on the structure of the gene product [30].

The overall objective of this study was to gain insights into the pathobiology of IA by examining the chromatin features in genetic regions known to confer risk for aneurysm within pathologically relevant cells. Our secondary objective was to determine if genetic variation in any of these regions could affect gene expression differences reported in our previous neutrophil transcriptome profiling study [6]. To do this, we investigated IA-associated genetic risk loci (validated in at least two studies) reported by the meta-analysis by Alg et al. [25] to determine if they contained (A) functional, regulatory elements, such as histone modifications; (B) genes that are relevant to vascular and immune or inflammatory function that could be involved in IA; and (C) differentially expressed genes identified in our previous study [6]. To investigate the epigenetic effects of SNPs in *specific cell types*, in this study, we separately examined the loci in human umbilical vein endothelial cells (HUVECs), monocytes, neutrophils, and peripheral blood mononuclear cells (PBMCs) using data available from the Encyclopedia of DNA Elements (ENCODE) project [31]. Specifically, we assessed the presence of H3K4me1/H3K27ac histone marks, CCCTC-binding factor (CTCF) sites, and transcription-factor binding sites (TFBSs). Furthermore, we examined whether genetic variation might impact other epigenetic features, such as DNA methylation, by querying DNA methyl-seq data collected by Yu et al. and if genetic variation could affect gene expression differences reported in the aneurysmal tissue in Yu et al. and circulating immune cells reported by Tutino et al. [6, 32].

## Methods

### Defining LD blocks

The identities of IA-risk SNPs were obtained from a comprehensive, published meta-analysis by Alg et al. [25], which evaluated 66 case-controlled studies and identified 19 significant IA-associated SNPs [25]. Importantly, all 19 identified SNPs had been reported as significantly associated with IA in *two or more* publications. We note, however, that these publications did not all make a distinction of whether the IAs were saccular or fusiform, and thus the 19 SNPs may be related to either type of IA, or both.

It should be noted that SNPs identified on GWAS do not identify the causal polymorphism; rather, they index a larger genetic region where risk may operate, and thus the causal SNP may be anywhere within the LD block. For this reason, it was critical for us to examine the haplotype blocks surrounding the index SNPs of interest. The SNP Annotation and Proxy (SNAP) search tool [33] and the proxy search within Single Nucleotide Polymorphisms Annotator (SNiPA) tool were used to identify linkage disequilibrium (LD) (haplotype) blocks associated with each SNP. We used the following settings for SNAP: SNP dataset – 1000 genome pilot 1 and HapMap3 (release 2); r^2^ threshold – 0.9; population panel – CEU; distance limit – 500. And the following settings for SNiPA: genome assembly – GRCh37; variant set – 1000 Genomes; population – American; genome annotation – Ensembl 87; r^2^ threshold – 0.9. The smallest and largest genomic positions were used as the start and stop locations, respectively, for each LD block.

### Identification of H3K4me1/H3K27ac histone marks within LD blocks

We queried genomic regions that might have enhancer function by identifying H3K4me1 and H3K27ac histone marks [34]. ENCODE data was used for genomic locations of H3K4me1/H3K27ac marks in HUVECs, monocytes (CD14+ RO01746), and peripheral blood mononuclear cells (only H3K4me1 data were available). We used H3K4me1/H3k27ac ChIP-Seq data from healthy adult neutrophils reported by Jiang et al. [35] to find H3K4me1/H3K27ac genomic locations in neutrophils (GEO:GSE66896). ENCODE histone data were downloaded from the University of California Santa Cruz Genome Browser ENCODE database [36] with the following accession numbers: HUVECs H3K4me1 GSM733690, HUVECs H3K27ac GSM733691, monocytes H3K4me1 GSM1003535, monocytes H3K27ac GSM1003559, and PBMCs H3K4me1 GSM788084.

To identify the intersection of H3K4me1/H3K27ac peaks within LD regions, we used the BEDTools software intersect command [37], following the procedure established in Jiang et al. [35]. In brief, 10,000 random regions in the human genome of the average length (32,312 bp) of SNAP assessed IA LD blocks were generated in BEDTools using the random intersect command. BEDTools intersect was used to determine (a) the number of LD regions that overlap with histone peaks, (b) the number of random regions that overlap with histone peaks, (c) the number of LD regions that do not overlap with histone peaks, and (d) the number of random regions that do not overlap with histone peaks. To determine whether H3K4me1/H3K27ac marks within IA LD blocks occurred at a statistically-greater-than-expected frequency, we performed a Fisher’s exact test (*p*-value< 0.05 was considered significant).

### Identification of CTCF binding sites within LD blocks

We investigated CTCF sites within IA-risk LD blocks using ENCODE data for HUVECs and monocytes (data not available for other cell types) as an indicator of chromatin organization that could affect gene expression. CTCF data were downloaded under the following accession numbers: HUVECs GSM733716, monocytes GSM1003508. As conducted for histone marks, BEDTools intersect was used to determine the intersection of binding sites within LD regions and randomly generated regions. A *p*-value< 0.05 (Fisher’s exact test) was used to determine LD regions with significant CTCF sites.

### Identification of TF binding sites within LD blocks

An important feature of functional elements in the noncoding genome (including enhancers) is the presence of multiple TFBSs within LD blocks. To determine whether H3K4me1/H3K27ac-marked regions were functional, we assessed the presence of TF binding sites within these regions. HUVECs were the only cell type considered because they were the only ones to have significant histone modifications in the IA-risk LD blocks. All TFs with data available for HUVECs within the ENCODE data sets were considered. Binding sites for EZH2, FOS, GATA2, JUN, MAX, MYC, POLR2A transcription factors were queried within the histone-marked locations of the IA-associated LD blocks. ENCODE data were downloaded for the following accession numbers: EZH2 wgEncodeEH003084; FOS wgEncodeEH001774; GATA2 wgEncodeEH001758; JUN wgEncodeEH000719; MAX wgEncodeEH000768; MYC wgEncodeEH000561; POLR2A wgEncodeEH000061, wgEncodeEH000552, wgEncodeEH000702, wgEncodeEH002297, wgEncodeEH002298. Sites were defined as “promoters” if they fell within 5 kb upstream or 1 kb downstream of the transcription start site; sites outside that window were considered distal. The transcription start site used to define TF as a promoter or distal site was provided by Switchgear Genomics on the University of California Santa Cruz Genome Browser [38].

### Identifying molecular pathways of genes within extended LD regions

Genes that fell within 200 kb upstream or downstream of the IA-associated SNPs were input to the Database for Annotation, Visualization and Integrated Discovery (DAVID) [39, 40] (https://david.ncifcrf.gov/home.jsp, accessed March 2019). This tool uses a large knowledge base to identify associated biological processes and pathways for given sets of genes. We used the default settings for our analysis. We also implemented an alternative method of gene ontology analysis via the Gene Ontology Term Finder (GO::TermFinder) [41] (https://go.princeton.edu/cgi-bin/GOTermFinder, accessed March 2019). We used genes that fell within 200 kb upstream or downstream of the IA-risk SNPs as the input gene list. GO::TermFinder assessed whether the input gene list was enriched for any specific gene ontology term to a greater degree than what would be expected by chance (q-value< 0.05). Default settings for GO::TermFinder were used to generate molecular function, biological process, and cellular component ontologies.

### Analysis of differential methylation and gene expression within LD blocks

We also evaluated genetic regulation of gene expression using DNA methylation data produced in the study conducted by Yu et al. [32], which is reported under the accession number of GSE75434. These authors compared DNA methylation within IA tissue samples to superficial temporal artery (STA) tissue from the same individual using the Infinium HumanMethylation450 BeadChip Kit (Illumina, San Diego, California). We determined whether any of these differentially methylated regions overlapped with the IA-risk LD blocks.

Yu et al. [32] also characterized the gene expression profiles of these IA and STA tissue samples using the Human Genome U133 Plus 2.0 GeneChip microarray (Affymetrix, Santa Clara, California). Raw microarray data, available under the accession number of GSE75436, were normalized by applying robust multichip average (RMA) normalization [42] in R with STA as control group. Genes with a fold-change of > 2 and a false discovery rate (FDR) of < 0.05 (after applying John Storey multiple hypothesis correction [43] to *p*-values calculated by an F-test of control and IA groups) were considered to be differentially expressed genes (DEGs). We identified the chromosomal locations for these genes in R using the human genome library [44], which draws from NCBI’s Entrez Gene database (https://www.ncbi.nlm.nih.gov/gene) and determined whether any of these genes fell within any of the IA-associated LD blocks (identified from Alg et al. [25]). Similarly, we examined the set of differentially expressed genes (*p*-value< 0.05, fold-change≥2) identified in our previous study that compared gene expression of circulating neutrophils from individuals with and without IAs (GSE106520) [6]. We evaluated whether there was agreement between those differentially expressed genes and the IA-risk LD blocks.

For all three data sets of interest (Yu et al. [32] methylation, Yu et al. [32] gene expression, and Tutino et al. [6] gene expression) the intersect command of BEDTools was used to determine whether any overlap existed between IA-risk LD blocks and the regions of interest generated from these additional data sets. The random regions that approximate background genome previously used to assess the significance of histone marks and CTCF sites were again used to determine significance of any overlap with regions of interest. A *p*-value< 0.05 (Fisher’s exact test) was used to determine significance.

## Results

### Queried LD blocks

From the 19 IA-risk SNPs identified in a comprehensive meta-analysis by Alg et al. [25], we were able to identify LD blocks for 16 of these SNPs, as given in Table 1. For the remaining 3 SNP loci (9p21.3 rs1333040, *CSPG2* rs173686, *ACE* I/D), there was insufficient information available through SNAP search tool to assess linkage disequilibrium blocks. As evident in Table 1, the majority (11) of the IA-associated SNPs fall within non-coding regions of the genome, which is typical of complex traits. There are 4 SNPs within the set we examined that fall within an exon; however, this does not necessarily mean that the SNPs alter the coding function of that gene [45].

**Table 1:** Positional information for 16 IA-risk single nucleotide polymorphisms and the associated linkage disequilibrium blocks^a^

SNP	LD Block	SNP Location	Nearest Gene in LD Block
rs4934	chr14:95078677–95,080,803	Exonic	*SERPINA3*
rs42524	chr7:94043239–94,049,356	Exonic	*COL1A2*
rs1132274	chr20:17594030–17,600,114	Exonic	*RRBP1*
rs1800255	chr2:189841613–189,867,882	Exonic	*COL3A1*
rs1429412	chr2:198148191–198,223,121	Intergenic	*ANKRD44*
rs6841581	chr4:148365339–148,414,651	Intergenic	*EDNRA*
rs9298506	chr8:55421614–55,462,324	Intergenic	–
rs10757278	chr9:22077085–22,125,503	Intergenic	*CDKN2B-AS1*
rs10958409	chr8:55309731–55,328,116	Intergenic	–
rs251124	chr5:82805424–82,826,254	Intronic	*VCAN*
rs700651	chr2:198541398–198,631,714	Intronic	*BOLL*
rs1800796	chr7:22766246–22,771,738	Intronic	*IL6*
rs2891168	chr9:22072264–22,125,503	Intronic	*CDKN2B-AS1*
rs3767137	chr1:22160723–22,168,310	Intronic	*HSPG2*
rs4628172	chr7:15493884–15,506,529	Intronic	*AGMO*
rs6538595	chr12:95489131–95,516,843	Intronic	*FGD6*

^a^SNPs reported by Alg et al. [25] were mapped to LD blocks using SNAP and SNiPA tools. The UCSC Genome Browser was used to visually determine SNP location and nearest gene

Abbreviations: *chr* Chromosome, *LD* Linkage disequilibrium, *rs* Reference SNP cluster ID, *SNP* Single nucleotide polymorphism, “–” = no gene)

### Location of H3K4me1/H3K27ac marks within LD blocks

To evaluate evidence of enhancer function within the IA-risk LD blocks, we used ENCODE data to determine whether H3K4me1 and H3K27ac marks expressed in HUVECs (relevant to endothelial biology) or monocytes, neutrophils, and PBMCs (relevant to inflammatory biology) were enriched within the LD blocks, compared to the genome background. HUVECs exhibited the greatest number of histone marks within the IA-risk LD blocks as compared to the randomly generated regions that represent the background genome. Table 2 shows that H3K4me1 enrichment was found in 15 blocks, while 10 of those blocks also exhibited H3K27ac marks. Both H3K4me1 and H3K27ac marks were statistically significantly enriched in LD blocks when compared to the genome background as assessed by Fisher’s exact test (*p* = 4.7E-05, *p* = 0.012; respectively). When we examined neutrophil, monocyte, and PBMC data, we did not find significant enrichment for H3K4me1 or H3K27ac marks within the IA-associated risk loci above the background genome levels.

**Table 2:** Histone marks present in IA-associated linkage disequilibrium blocks^a^

LD Block	SNP	HUVEC		M0		PNL		PBMC
		H3K4me1^b^	H3K27ac ^b^	H3K4me1	H3K27ac	H3K4me1	H3K27ac	H3K4me1
chr1:22160723–22,168,310	rs3767137	Y	N	N	N	N	N	N
chr2:189841613–189,867,882	rs1800255	Y	Y	N	N	N	N	N
chr2:198148191–198,223,121	rs1429412	Y	Y	Y	Y	Y	Y	Y
chr2:198541398–198,631,714	rs700651	Y	Y	Y	Y	Y	Y	Y
chr4:148365339–148,414,651	rs6841581	Y	N	N	N	N	N	N
chr5:82805424–82,826,254	rs251124	Y	Y	Y	Y	N	N	Y
chr7:15493884–15,506,529	rs4628172	Y	N	N	N	N	N	N
chr7:22766246–22,771,738	rs1800796	Y	Y	Y	Y	Y	N	Y
chr7:94043239–94,049,356	rs42524	Y	N	N	N	N	N	N
chr8:55309731–55,328,116	rs10958409	Y	Y	N	N	N	N	N
chr8:55421614–55,462,324	rs9298506	Y	Y	N	N	N	N	N
chr9:22072264–22,125,503	rs2891168	Y	Y	Y	N	N	N	Y
chr9:22077085–22,125,503	rs10757278	Y	Y	Y	N	N	N	Y
chr12:95489131–95,516,843	rs6538595	Y	Y	Y	Y	Y	N	Y
chr14:95078677–95,080,803	rs4934	N	N	N	N	N	N	N
chr20:17594030–17,600,114	rs1132274	Y	N	Y	Y	Y	Y	Y

^a^“Y” indicates histone mark for given cell type was present within that LD block. Marks that occur at statistically greater than expected frequency are denoted with ^b^. Abbreviations: *LD* Linkage disequilibrium, *chr* Chromosome, *rs* Reference SNP cluster ID, *SNP* Single nucleotide polymorphism, *Y* Yes, *N* No, *HUVEC* Human umbilical vein endothelial cell, *M0* Monocyte, *PNL* Polymorphonuclear leukocyte - neutrophil, *PBMC* Peripheral blood mononuclear cell

**Fig. 1:**
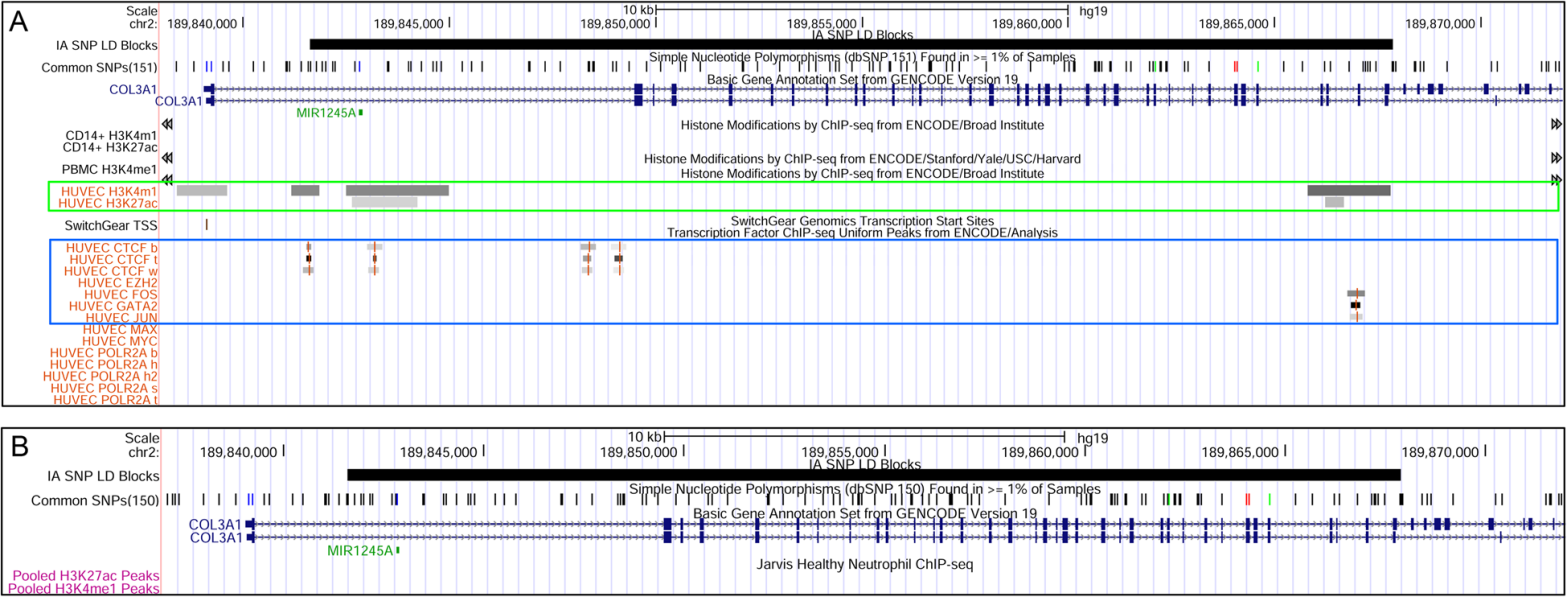
**a** UCSC Genome Browser screenshot of epigenetic landscape around SNP rs1800255 within the exon of *COL3A1*. Black horizontal bar at top represents the LD block of the associated IA-risk SNP. The next tracks in descending order within panel A are the H3K4me1 and H3K27ac peak regions generated from ChIP-Seq data for monocytes (CD14+), PBMCs, and HUVECs. Gray bars in the lower section represent transcription factor ChIP-Seq of 8 factors from ENCODE with data available for HUVECs. Notice that *COL3A1* is expressed in this LD block. Histone marked regions, represented by the gray bars in the middle of this panel, are only present for HUVECs and encompass multiple TFBSs. **b** Tracks for neutrophil H3K4me1 and H3K27ac peak regions generated from ChIP-Seq data. *Key:* UCSC=University of California Santa Cruz, SNP = single nucleotide polymorphism, COL3A1 = collagen type III alpha chain a, LD = linkage disequilibrium, IA = intracranial aneurysm, PBMC = peripheral blood mononuclear cell, HUVEC = human umbilical vein endothelial cell, ChIP-Seq = chromatin immunoprecipitation-sequencing, TFBS = transcription factor binding site; ENCODE = Encyclopedia of DNA [deoxyribonucleic acid] Elements

**Fig. 2:**
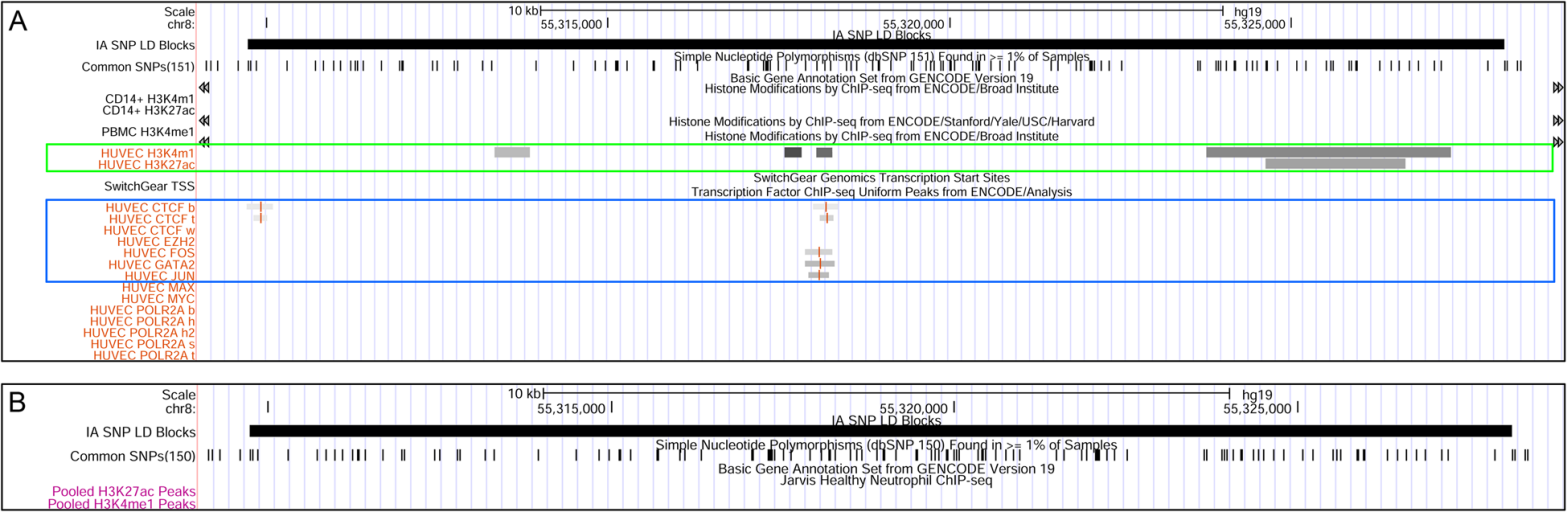
**a** UCSC Genome Browser screenshot of epigenetic landscape around intergenic SNP rs10958409. Black horizontal bar at the top represents the LD block of the associated IA-risk SNP. The next tracks are for monocyte (CD14+), PBMC, and HUVEC H3K4me1 and H3K27ac peak regions generated from ChIP-Seq data. Gray bars in the lower section of panel A represent transcription factor ChIP-Seq of 8 factors from ENCODE with data available for HUVECs. Histone marked regions, represented by the gray bars in the middle of panel A, are only present for HUVECs and encompass multiple TFBS. **b** Tracks for neutrophil H3K4me1 and H3K27ac peak regions generated from ChIP-Seq data. *Key:* UCSC=University of California Santa Cruz, SNP = single nucleotide polymorphism, LD = linkage disequilibrium, IA = intracranial aneurysm, PBMC = peripheral blood mononuclear cell, HUVEC = human umbilical vein endothelial cell, ChIP-Seq = chromatin immunoprecipitation-sequencing, TFBS = transcription factor binding site). ENCODE = Encyclopedia of DNA [deoxyribonucleic acid] Elements

Figure 1 shows the landscape around the SNP, rs1800255, within the 30th exon of the collagen type III alpha chain (*COL3A1*). This gene encodes for components of type III collagen, an integral component of blood vessel walls. Mutations in this gene are known to cause the vascular type of Ehlers-Danlos syndrome (type IV), a genetic disease affecting connective tissue. Ehlers-Danlos syndrome is associated with increased presence of IA; 12% of those with Ehlers-Danlos have IA [46], which is approximately double the rate of that in the general population [47]. Furthermore, only ECs express histone marks in this LD block, indicated by the green box. In this LD block, it is unclear whether the genetic risk operates through the protein-coding gene or through enhancer activity within the ECs. Figure 2 shows the landscape of the LD block containing rs10958409, an intergenic SNP; note that there are no protein-coding genes within this haplotype. In this LD block, as in Fig. 1, we note that only the ECs show enrichment for H3K4me1/H3K27ac histone marks (green box). In this case, it is likely that genetic risk impinges on the endothelium by enhancer regulation.

### CTCF binding sites within LD blocks

CTCF is an important regulator of three-dimensional chromatin architecture and therefore gene expression [48, 49]. We investigated if CTCF binding sites were present in HUVECs and monocytes within the IA-risk LD blocks (CTCF binding site data was not available for neutrophils or PBMCs). As shown in Table 3, HUVECs exhibited CTCF binding sites within 10 LD blocks, 8 of which also had both H3K4me1 and H3K27ac histone marks (see also Table 2). CTCF binding sites were found in 7 LD blocks for monocytes, 5 of which corresponded to blocks with both histone marks (see also Table 2). The frequency of CTCF binding sites within LD blocks was not significantly greater than the frequency of CTCF binding sites within the randomly generated regions (Fisher’s exact test). In Figs. 1a and 2a, we noticed CTCF sites within the IA-associated LD blocks (first 3 rows within the blue box).

**Table 3:** CTCF binding sites present in IA-associated linkage disequilibrium blocks for endothelial cells and monocytes^a^

LD Block	SNP	HUVEC	M0
chr1:22160723–22,168,310	rs3767137	Y	Y
chr2:189841613–189,867,882	rs1800255	N	N
chr2:198148191–198,223,121	rs1429412	Y	Y
chr2:198541398–198,631,714	rs700651	Y	Y
chr4:148365339–148,414,651	rs6841581	N	N
chr5:82805424–82,826,254	rs251124	Y	Y
chr7:15493884–15,506,529	rs4628172	N	N
chr7:22766246–22,771,738	rs1800796	Y	Y
chr7:94043239–94,049,356	rs42524	N	N
chr8:55309731–55,328,116	rs10958409	Y	N
chr8:55421614–55,462,324	rs9298506	Y	N
chr9:22072264–22,125,503	rs2891168	N	N
chr9:22077085–22,125,503	rs10757278	Y	Y
chr12:95489131–95,516,843	rs6538595	N	N
chr14:95078677–95,080,803	rs4934	Y	N
chr20:17594030–17,600,114	rs1132274	Y	Y

^a^ “Y” indicates CTCF binding site for given cell type was present within that LD block. Data were unavailable for neutrophils and peripheral blood mononuclear cells. Abbreviations: *CTCF* CCCTC binding factor site, *LD* Linkage disequilibrium, *chr* Chromosome, *rs* Reference SNP cluster ID, *SNP* Single nucleotide polymorphism, *Y* Yes, *N* No, *HUVEC* Human umbilical vein endothelial cell, *M0* Monocyte

### TF binding sites within histone marks of HUVEC LD blocks

Although the H3K4me1 and H3K27ac histone marks suggest poised and active enhancers, respectively, a TFBS within the histone-marked regions that fall within the IA-associated LD blocks strongly indicates that the region is functional and can ultimately transcribe RNA [50]. Because the IA-associated LD blocks were enriched for H3K4me1/H3K27ac marks just in HUVECs, we only examined TFBSs in the histone-marked sections for HUVECs. We examined all transcription factors with data available for HUVECs in ENCODE. Figure 1a and Fig. 2a also provide examples of TFBS that fall within the histone-marked regions (blue box). Binding sites for EZH2, FOS, GATA2, JUN, MAX, MYC, and/or POLR2A were present within the H3K4me1/H3K27ac-marked regions as shown in Table 4.

**Table 4:** TF binding sites present within histone-marked regions of IA-associated LD blocks for endothelial cells^a^

LD Block	SNP	Distal TF	Promoter TF
chr1:22160723–22,168,310	rs3767137	–	POLR2A
chr2:189841613–189,867,882	rs1800255	FOS, GATA2, JUN	–
chr2:198148191–198,223,121	rs1429412	FOS, GATA2, JUN	–
chr2:198541398–198,631,714	rs700651	FOS	MAX^b^, MYC^b^, POLR2A^b^
chr4:148365339–148,414,651	rs6841581	–	EZH2
chr5:82805424–82,826,254	rs251124	JUN^b^	–
chr7:15493884–15,506,529	rs4628172	FOS	–
chr7:22766246–22,771,738	rs1800796	FOS^b^, GATA2^b^, JUN^b^, POLR2A^b^	POLR2A^b^
chr7:94043239–94,049,356	rs42524	FOS, JUN	–
chr8:55309731–55,328,116	rs10958409	FOS, GATA2, JUN	–
chr8:55421614–55,462,324	rs9298506	–	–
chr9:22072264–22,125,503	rs2891168	FOS^b^, GATA2^b^, JUN^b^, POLR2A^b^	–
chr9:22077085–22,125,503	rs10757278	FOS^b^, GATA2^b^, JUN^b^, POLR2A^b^	–
chr12:95489131–95,516,843	rs6538595	FOS^b^, GATA2^b^, JUN^b^, POLR2A^b^	–
chr14:95078677–95,080,803	rs4934	–	–
chr20:17594030–17,600,114	rs1132274	–	–

^a^ TF binding site identification only performed for HUVEC. All TFs were present within H3K4me1-marked regions of IA-associated LD blocks. TFs denoted with ^b^ were present in regions of IA-associated LD blocks marked by both H3K4me1 and H3K27ac histone modifications. (*IA* Intracranial aneurysm, *LD* Linkage disequilibrium, *chr* Chromosome, *SNP* Single nucleotide polymorphism, *HUVEC* Human umbilical vein endothelial cell, “–” = no TF)

Table 4 also shows the location of the TFBS with respect to the transcription start site and promoter or distal elements. Distal TFBSs may be involved in regulatory processes in regions other than promoters, such as intergenic regions where noncoding RNA is encoded [51]. We noted significant TF binding in distal regions of the IA-associated LD blocks. There were distal TFBSs for FOS in 10 of the 16 LD blocks, for JUN in 9 of the 16 LD blocks, and for GATA2 in 7 of the 16 LD blocks. POLR2A had distal or promoter TFBSs in 6 of the 16 LD blocks. Furthermore, multiple TFBS were evident in the promoter regions within the IA-associated LD blocks. EZH2, MAX, MYC, and POLR2A were present in the promoter region for at least 1 of the LD blocks of interest. These TFs interact together and with another transcription factor (MAD) to activate or inhibit specific gene transcription and affect cell proliferation, differentiation, and death [52]. The overlap of histone modifications and promoter TF binding sites within the IA-risk regions could suggest a complicated interaction between enhancers and promoters that can affect gene regulation.

### Enriched molecular pathways of disease-associated SNPs

We used the DAVID database to identify significantly enriched biological ontology terms for the 67 protein-coding genes located ±200 kb from the IA-associated SNPs. Of these, 57 corresponded to DAVID IDs and were used in the classification. Twenty ontologies were identified as significant (Benjamini Hochberg-adjusted *p*-value < 0.05) using the functional annotation tool, as shown in Table 5. These ontologies were functionally related to the serpin family, extracellular matrix (ECM) organization, receptor interactions, and fibrillar collagen.

**Table 5:** DAVID Functional annotation results^a^

Term	Source	No. of genes	*P*-Value	Q-Value
Serpin domain	INTERPRO	7	4.30E-10	4.90E-08
Serpin family	INTERPRO	7	4.30E-10	4.90E-08
SERPIN	SMART	7	9.00E-10	3.30E-08
Serine protease inhibitor	UP_KEYWORDS	7	4.20E-08	5.50E-06
Serine-type endopeptidase inhibitor activity	GOTERM_MF_DIRECT	7	1.80E-07	1.80E-05
Protease inhibitor	UP_KEYWORDS	7	3.70E-07	2.50E-05
Negative regulation of endopeptidase activity	GOTERM_BP_DIRECT	6	1.20E-05	4.80E-03
Extracellular matrix structural constituent	GOTERM_MF_DIRECT	5	2.70E-05	1.40E-03
Protease inhibitor I4, serpin, conserved site	INTERPRO	4	7.40E-05	4.20E-03
Extracellular space	GOTERM_CC_DIRECT	13	9.30E-05	1.00E-02
Extracellular matrix	GOTERM_CC_DIRECT	7	9.50E-05	5.20E-03
Extracellular matrix organization	GOTERM_BP_DIRECT	6	1.20E-04	2.40E-02
Propeptide: C-terminal propeptide	UP_SEQ_FEATURE	3	1.40E-04	3.80E-02
Domain: Fibrillar collagen NC1	UP_SEQ_FEATURE	3	2.80E-04	3.70E-02
Extracellular matrix	UP_KEYWORDS	6	3.00E-04	1.30E-02
Fibrillar collagen, C-terminal	INTERPRO	3	3.30E-04	1.20E-02
Secreted	UP_KEYWORDS	14	3.30E-04	1.10E-02
Skeletal system development	GOTERM_BP_DIRECT	5	3.70E-04	4.70E-02
Ehlers-Danlos syndrome	UP_KEYWORDS	3	4.60E-04	1.20E-02
COLFI	SMART	3	4.70E-04	8.60E-03

^a^Functional annotation results returned from DAVID using genes within ±200 kb of intracranial aneurysm-associated linkage disequilibrium blocks. Abbreviations: *DAVID* Database for Annotation, Visualization and Integrated Discovery; *No*. Number

**Fig. 3:**
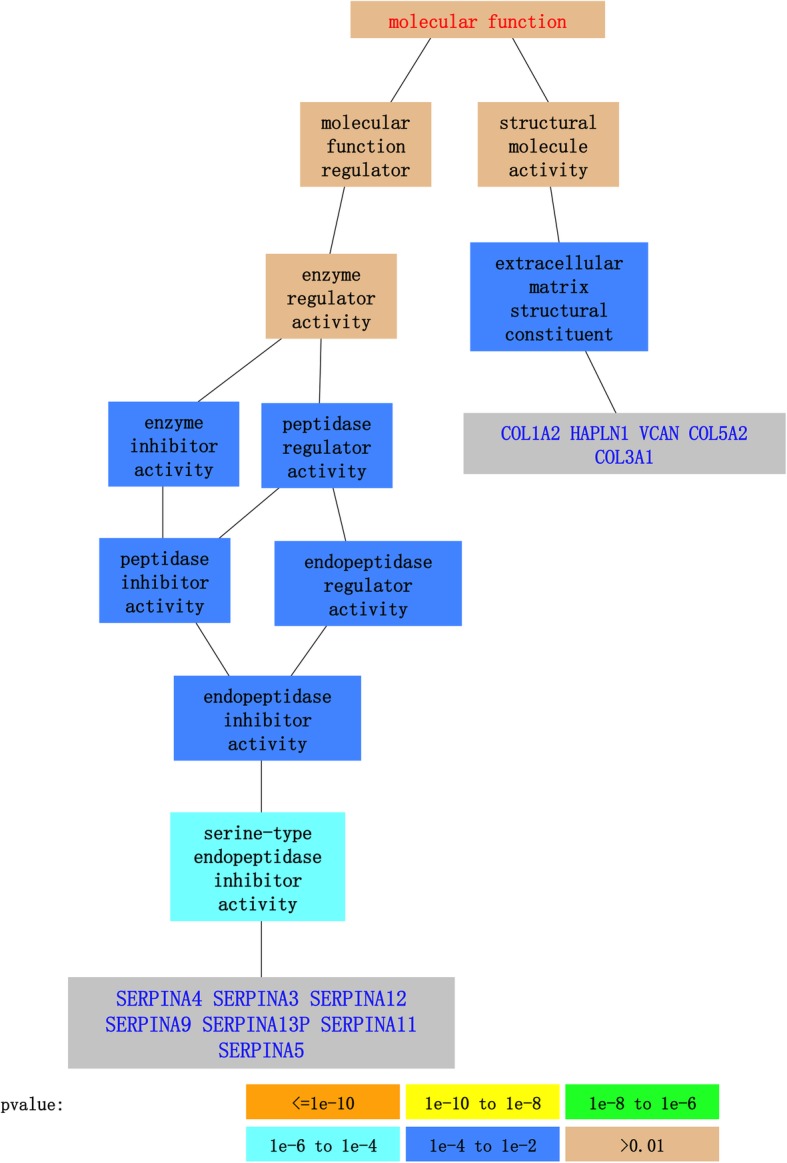
Gene Ontology term finder molecular function results for genes within extended IA-associated LD blocks. Blocks in blue represent significant ontologies

We also conducted gene ontology analysis using GO::TermFinder to identify significant (Benjamini Hochberg-adjusted *p*-value < 0.05) molecular processes, functions, and components enriched in the list of 67 genes. The significant molecular function terms and their associated *p*-values are shown in Fig. 3 (excluding “unannotated” terms). Significant biological functions, processes, and components included ontologies such as endopeptidase regulator activity, regulation of endopeptidase activity, ECM structural constituent, fibrillar collagen trimer, and complex of collagen trimers. The entire list of significant ontologies is presented in Additional file 1: Table S1. Flow diagrams of significant molecular processes and components are presented in Additional file 1: Figures S1 and S2, respectively.

### Differential methylation and expression within LD blocks

To evaluate if differentially methylated regions could be affecting gene regulation within the IA-risk LD blocks, we compared the differentially methylated regions reported by Yu et al. [32] to those of the 16 identified LD blocks. Yu et al. [32] identified 11,022 differentially DNA methylated regions when they compared IA tissue samples and matched STA tissue samples. Table 6 reports the 4 differentially methylated sites that intersected with 3 LD blocks associated with risk for IA; however, this was not statistically significant when compared to intersections between differentially methylated regions and randomly-created background genome regions.

**Table 6:** Differentially methylated regions present within IA-associated linkage disequilibrium blocks^a^

LD Block	SNP	Differentially Methylated Region
chr2:198148191–198,223,121	rs1429412	chr2:198173194–198,173,317
chr4:148365339–148,414,651	rs6841581	chr4:148413287–148,413,410
chr20:17594030–17,600,114	rs1132274	chr20:17595355–17,595,478
		chr20:17595448–17,595,571

^a^Differentially methylated regions identified in tissue study by Yu et al. (32)

Abbreviations: *chr* Chromosome, *LD* Linkage disequilibrium, *rs* Reference SNP cluster ID, *SNP* Single nucleotide polymorphism

We also analyzed gene expression in vascular tissue to determine if the 16 LD blocks could affect the expression differences reported in the literature. To that end, data from two sources was used. First, we used the differential RNA expression data from IA tissue and matched STA tissue from Yu et al. [32]. We generated a list of 596 significant probes (fold change> 2, FDR < 0.05), which corresponded to 516 gene transcripts. There was not sufficient data available through the Entrez database to determine the location for 40 of the transcripts; consequently, these transcripts were excluded from the analysis. Ultimately, 476 regions were input to BEDTools for comparison with IA-risk LD blocks. Only 1 gene, *COL1A2*, overlapped with the IA-risk blocks, and this result was not found to be statistically significant by Fisher’s exact test.

Secondly, we performed a similar analysis using RNA expression differences reported in inflammatory cells from our previous RNA sequencing study profiling circulating neutrophils of individuals with and without IA [6]. Of the 82 genes that showed differential expression between patients with IA and those without, we were able to assess chromosomal positions for 76 transcripts. None of these 76 genes were located within the LD blocks associated with IA risk. This finding suggests that the IA-risk loci may (a) act on the coding sequences (and therefore function) of one or more of the genes within the haplotypes, (b) act by long-distance enhancer-promoter interactions, or (c) both.

## Discussion

In this study, we used ENCODE data to investigate epigenetic landscapes of 16 prominent IA risk haplotypes. Within these haplotype blocks, we found evidence that genetic alterations (i.e. SNPs) may affect transcription of genes relevant to IA development through modulation of enhancer activity. Our analyses showed that functional regulatory elements within the IA-associated risk regions were present to a greater degree in ECs than immune cells. This suggests that genetic risk for IA is more likely to be conferred through the ECs than immune cells. Differentially expressed transcripts that we previously identified in circulating neutrophils were not associated with the investigated LD blocks. Thus, the abnormal gene expression observed in circulating neutrophils may reflect the presence of the aneurysmal lesion, rather than genetic risk that could precede IA initiation.

### Genetic risk in the endothelium of persons with IA

The 16 IA-associated LD blocks considered in this study not only contained coding genes, but also contained apparent enhancers in noncoding regions that were specifically enriched in ECs. The histone marks H3K4me1 and H3K27ac (associated with poised or active enhancer function) were seen exclusively in ECs. H3K4me1 and H3K27ac marks in the IA-associated haplotypes were present at a greater-than-expected frequency in HUVECs (*p* = 4.7E-05 for H3K4me1 marks, *p* = 0.012 for H3K27ac marks) but not in monocytes, neutrophils, or PBMCs.

In HUVECs, the H3K4me1/H3K27ac-marked sites also demonstrated abundant TFBSs, corroborating the idea that these noncoding regions are indeed functional regulatory elements. CTCF binding sites were present within the IA-associated LD blocks in HUVECs, although not in a greater-than-expected frequency (*p* = 0.066). These results suggest that genetic risk for IA is more likely to operate on a vascular component of the disease (i.e., ECs) rather than an immune component, because there was no enrichment for these functional features in immune cells.

Our bioinformatics analyses of the coding elements within or adjacent to the 16 IA-risk LD blocks also suggests that genetic risk affects the endothelium. DAVID analysis demonstrates ontological terms significantly associated with these genes to be “extracellular matrix,” “collagen,” “Ehlers-Danlos syndrome,” and “serpin.” Our GO::TermFinder results echo these findings; significant molecular functions of these genes were related to endopeptidase activity/regulation (*SERPINA4*, *SERPINA3*, *SERPINA12*, *SERPINA9*, *SERPINA13P*, *SERPINA11*, and *SERPINA5*) and ECM structural components (*COL1A2*, *HAPLN1*, *VCAN*, *COL5A2*, and *COL3A1*).

Our results implicate ECs as the cell type to most likely be affected by genetic risk. ECs have long been recognized as key players in IA pathogenesis [53]. Animal models of IA initiation report that EC dysfunction is one of the first changes that correlate to aneurysm formation [54]. This is likely because ECs dynamically react to aberrant hemodynamics that occur in locations where IAs preferentially form, i.e., at bifurcation apices and outer curves of tortuous arteries [55]. Specifically, studies have shown that ECs react to increased wall shear stress by triggering signaling cascades that produce and activate proteases, namely matrix metalloproteinase (MMP)-2 and MMP-9, which damage the internal elastic lamina, cause smooth muscle cell (SMC) apoptosis, and weaken the vascular wall [56]. We hypothesize that aberrant EC response mechanisms, potentially arising from the SNPs we investigated, could propagate pathological remodeling and consequently aneurysm formation and growth. Endothelial dysfunction, particularly in the breakdown of EC continuity, could lead to inflammatory responses, both within the vessel wall and in circulating cells, which are observed in IAs [56, 57].

A further examination of the genes within the 16 LD blocks also shows how genetic risk could affect fundamental pathobiological mechanisms in IAs. Remarkably, 4 of the SNPs in this study fall within exons of *COL1A2*, *COL3A1*, ribosome-binding protein 1 (*RRBP1*), and *SERPINA3* and thus could exert their effects by directly changing the structure and/or function of the translated proteins. Polymorphisms in *COL1A2* and *COL3A1* (which together make up 80–90% of arterial collagens) are well-established in IAs [58, 59], and are known to lead to Ehlers-Danlos syndrome [13, 60, 61]. Production of defective collagens by ECs could contribute to impairment of the function and integrity of the endothelial layer during IA formation [62]. However, because vascular SMCs produce *COL1* and *COL3* [63, 64], polymorphisms in the coding regions of these genes may reflect more extensive wall dysfunction due to their effect in vascular SMCs. SNPs in members of the serpin family could also affect ECM remodeling and stability, as they are inhibitors of serine proteases, which can degrade the matrix during IA formation [65]. In an animal model contrasting ruptured and unruptured aneurysms, Kataoka et al. [66] found neutrophil-derived cathepsin G expressed in walls of ruptured aneurysms. The main inhibitory target of *SERPINA3* is cathepsin G [67], which can activate pro-MMP-9 [68] and contribute to ECM remodeling [69, 70]. Therefore, we suspect that the mutations in *COL1A2, COL3A1,* and *SERPINA3* could lead to increased degradation and insufficient ECM repair during IA formation [56]. Conversely, the exonic SNP in *RRBP1*, a ribosome receptor, may trigger a more universal defect in protein expression, as poor interactions between the ribosome and endoplasmic reticulum could negatively affect protein processing during IA development [71].

The remaining SNPs we studied fell within noncoding regions of the genome; and thus, the influence of the IA-risk SNP is likely felt through the modulation of enhancer activity, CTCF binding sites, or TFBSs. These SNPs could affect RNA expression by modulating the transcription machinery’s activity for genes within the haplotype. Our data demonstrate that genes potentially affected by the SNPs in noncoding regions may also be biologically relevant to IA pathogenesis. Genes affected by these SNPs could play a role in EC regulation of vascular remodeling and signaling during IA. For example, endothelin receptor type A (*EDNRA*) encodes for the receptor of endothelin-1, which through binding to the receptor leads to vasoconstriction during maintenance of vascular homeostasis [72]. Endothelin-1 also stimulates inflammatory responses and proliferation [73–75], so altering the receptor *EDNRA* could also impact these key processes of aneurysmal remodeling. Additionally, *ANKRD44* is a subunit of protein phosphatase 6, which can inhibit activation of NF-κB [76], an important pathway in pro-inflammatory EC signaling during IA pathogenesis [77]. *IL6*, a proinflammatory cytokine, is secreted by both M1 macrophages [78] and lymphocytes [79] during IA formation and growth. Increased levels have been found in ruptured IAs during surgical clipping [80]. Individuals with this polymorphism have higher *IL6* plasma levels [81] and a higher risk of IA [82].

Interestingly, the analysis of methyl-seq data from Yu et al. [32] showed differential methylation in the LD blocks containing *EDNRA* and *ANKRD44*, suggesting that changes in their expression may be through an epigenetic mechanism. Additionally, alterations in the expression of genes such as *VCAN* and *HSPG2*, which encode proteins involved in cell adhesion, may affect the ability of circulating immune cells to adhere to the endothelium [83–85]. Several genes within the LD blocks of the SNPs that we studied (namely, *CDKN2B-AS1*, *AGMO*, *BOLL*, and *FGD6*) are not well characterized and warrant further investigation to determine how they may be associated with IA risk.

### Relating these findings to previous studies

Although direct experimental investigation of epigenetic landscapes in IA has been sparse, in a recent study, Laarman et al. [86] performed ChIP-seq on DNA from postmortem human Circle of Willis tissue to identify histone H3K4me1 and H3K27ac modifications in regulatory regions (distal enhancers and active promoters). They then queried if these regions overlapped with 19 known IA-associated SNP regions (from [22, 23]) and found that 7 of them overlapped with active regulatory regions. Three of the SNPs they queried were also investigated in the current study, namely, rs1132274 (on chr20), rs6841581 (on chr4), and rs658595 (on chr12). Interestingly, rs6841581 was found by Laarman et al. [86] to likely affect TF binding while rs1132274 and rs653859 had minimal and no predicted TF activity, respectively. In a follow-up study, Laarman et al. [87] elegantly used chromatin conformation capture technology to identify enhancer targets of 4 known risk loci and confirmed intrinsic enhancer activity via an in vivo reporter assay. Two of the loci studied by Laarman et al. overlapped with those in our study, namely chr4:148365339–148,414,651 (rs6841584) and chr9:22077085–22,125,503 (rs10757278). These studies provide compelling experimental evidence of enhancer activity in regions reported in the present study, particularly for the SNPs mentioned above (rs6841584 and rs10757278). However, because Laarman et al. [86, 87] investigated DNA from multiple cell types (from whole Circle of Willis tissue), using their data to interpret our findings should be limited, pending confirmation through experimental validation in specific cell types, such as ECs, obtained from aneurysm tissue.

In addition to investigating if the chromatin landscape around IA-risk SNPs could inform us if aberrant transcriptomes detected in neutrophils precede or follow IA formation, we queried whether gene expression differences in recent aneurysm tissue studies could be related to the influence of SNPs within the 16 LD blocks. In a set of 516 differentially expressed genes between IA tissue and STA tissue taken from data published by Yu et al. [32], only *COL1A2* fell within an LD block, pointing to the importance of functional collagen expression in the pathogenesis of IA. We may not have found overlap with any other DEGs and the IA-associated LD blocks because the data derived from Yu et al. [32] came from RNA samples from whole tissue and not cell-line ENCODE data. Thus, others cells, (i.e. SMCs, inflammatory cells, fibroblasts) may contribute to the expression differences determined from tissue samples and add significant noise to the data. In the future, laser micro-dissected sections of IA tissue could isolate ECs to better correlate expression differences with the epigenetic landscapes.

It is noteworthy that the IA-associated LD blocks did not contain any of the differentially expressed genes identified in our neutrophil RNA profiling study [6]. This suggests that expression changes in circulating neutrophils during IA formation are a response to the existing aneurysmal lesion, rather than being an indication of IA risk. This is consistent with our published bioinformatics results [6], in which we found enriched leukocyte activation processes in the neutrophils, characterized by elevated levels of *CD1D*, *CD7*, *CD86*, *CD177*, and *VNN1* antigens in patients with IA. This evidence led us to hypothesize that circulating neutrophils are activated in the bloodstream by contact with the diseased IA tissue [88–90] or with cytokines and chemokines thought to be released from the IA tissue [91].

In the present study, none of the IA-associated LD blocks were significantly enriched for histone marks or CTCF binding sites in neutrophils, which further supports our hypothesis. In fact, our results predict that functional regulatory elements in the IA-associated risk regions are present more in ECs, suggesting that genetic risk for IA is more likely to be conferred through the ECs than the immune cells. This is further supported by the Gene Ontology data demonstrating endopeptidase activity/regulation and ECM structural components, which may play significant roles in ECs, rather than immune cells (neutrophils). These results suggest that aberrant expression observed in circulating immune cells of individuals with IA is a secondary response following IA formation and not an indicator of genetic risk for the disease, at least for the 16 SNPs that we investigated. Therefore, the expression changes in circulating neutrophils that we observed could be caused by contact with inflamed aneurysm tissue or activation by chemokines and cytokines released from the aneurysm [92].

### Limitations

One limitation of this study is that we focused on IA-associated SNPs identified in a single comprehensive meta-analysis by Alg et al. [25], thus excluding SNPs that have been reported in other studies. However, the SNPs identified in the meta-analysis were found in at least 2 studies that analyzed a large volume of cases in controlled populations; consequently, these SNPs have a high likelihood of being associated with IA. Second, in this study, we focused on HUVECs, monocytes, neutrophils, and PBMCs and did not include data from other cells types, most notably vascular SMCs, which we recognize are critical to IA pathogenesis. Unfortunately, there was not sufficient cell-type specific ENCODE data available for vascular SMCs for use in our study. We also are assuming that the results from HUVECs generalize to endothelial cells derived from arterial-based aneurysms though it has been shown ECs in different tissues have distinct expression profiles [93]. Third, we used genes within ±200 kb of IA associated SNPs for functional annotation and gene ontology analyses. However, it is likely that the relevant enhancers may regulate genes that are not within these regions, as demonstrated in the review by Kessler et al. [94]. To identify genes affected by the enhancers we identified in this study, we would need to examine the topologically associated domains (TADs) of the IA associated risk SNPs. These domains may better describe which regions of the genome form interactions and thereby affect gene expression. We plan to study the TADs encompassing the IA-risk SNPs in the future. Lastly, we cannot be certain whether a specific genetic variant operates through the gene function in more complex landscapes that also include enhancer marks or TF binding. For instance, although a SNP may fall within an exon of a gene with biological significance in an IA, this region may also contain prominent H3K4me1/H3K27ac marks and function as a so-called “exonic enhancer” [45]. In cases like this, the genetic variant could operate through the coding sequence function, the enhancer function, or both.

## Conclusions

In this data-driven study, we analyzed 16 regions of known genetic risk for IAs that were identified by large GWAS and found that IA-risk SNPs were likely affecting the expression of genes relevant to IA development through modulation of enhancer activity. Using data from the ENCODE project, we were able to show that functional regulatory elements within the IA-associated risk regions were present to a greater degree in ECs compared to immune cells. Ontology analyses performed on genes potentially affected by each SNP showed cellular processes and functions related to regulation of ECM and protease. Although these findings do not exclude immune or inflammatory mediators as important elements in IA pathogenesis, they imply that known genetic risk factors for IA are more likely affecting the vessel wall than the circulating inflammatory cells. These results shed further light on how IA-associated SNPs may affect IA pathogenesis and highlight the importance of investigating noncoding elements in cell-type specific genomes. Our results also imply that the transcriptomic differences we have previously detected in circulating neutrophils are a response to the presence of the IA lesion, rather than an indication of risk for the disease.

## Supplementary information

**Additional file 1: Table S1.** Entire list of significant ontologies from GO::TermFinder. **Fig. S1.** Gene Ontology term finder molecular process results for genes within extended intracranial aneurysm-associated linkage disequilibrium blocks. Blocks in blue represent significant ontologies. **Fig S2.** Gene Ontology term finder molecular component results for genes within extended intracranial aneurysm-associated linkage disequilibrium blocks. Blocks in blue represent significant ontologies.

## Data Availability

All datasets cited in this paper are publicly available. Links are provided within the manuscript text.

## Abbreviations

ChIP-Seq: Chromatin immunoprecipitation-sequencing
*COL1A*1: Collagen type I alpha 1 chain
*COL1A*2: Collagen type I alpha 2 chain
*COL3A1*: Collagen type III alpha chain a
*COL5A*2: Collagen type V alpha 2 chain
CTCF: CCCTC-binding factor
DAVID: Database for Annotation, Visualization and Integrated Discovery
DEG: Differentially expressed genes
EC: Endothelial cell
ECM: Extracellular matrix
*EDNRA*: Endothelin receptor type A
ENCODE: Encyclopedia of DNA Elements
EZH2: Enhancer of zeste homolog 2
FDR: False discovery rate
GO:TermFinder: Gene Ontology Term Finder
GWAS: Genome-wide association studies
HUVECs: Human umbilical vein endothelial cells
IA: Intracranial aneurysm
LD: Linkage disequilibrium
MAX: MYC associated factor X
methyl-seq: Methylation sequencing
MMP: Matrix metalloproteinase
MYC: MYC proto-oncogene
PBMCs: Peripheral blood mononuclear cells
POLR2A: RNA polymerase II
R-genes: Resistance genes
RMA: Robust multichip average
*RRBP1*: Ribosome-binding protein
SMC: Smooth muscle cell
SNAP: SNP Annotation and Proxy
SNP: Single nucleotide polymorphism
STA: Superficial temporal artery
TF: Transcription factor
TFBS: Transcription factor binding site
