# Supplementary material for: Epigenetic landscapes suggest that genetic risk for intracranial aneurysm operates on the endothelium
Source: BMC Med Genomics. 2019 Oct 30;12:149. doi: 10.1186/s12920-019-0591-7 (PMC6821037; doi:10.1186/s12920-019-0591-7)
Supplement: Supplementary file 1 — Additional file 1: Table S1. Entire list of significant ontologies from GO::TermFinder. Fig. S1. Gene Ontology term finder molecular process results for genes within extended intracranial aneurysm-associated linkage disequilibrium blocks. Blocks in blue represent significant ontologies. Fig S2. Gene Ontology term finder molecular component results for genes within extended intracranial aneurysm-associated linkage disequilibrium blocks. Blocks in blue represent significant ontologies. [file 12920_2019_591_MOESM1_ESM.doc]

**Additional files for:**

**Epigenetic Landscapes Suggest that Genetic Risk for Intracranial Aneurysm Operates on the Endothelium**

**Authors**: Kerry E. Poppenberg BS^1,2^, Kaiyu Jiang PhD^3^, Michael K. Tso MD PhD^1,4^, Kenneth V. Snyder MD PhD^1,4,5^, Adnan H. Siddiqui MD PhD^1,4,5^, John Kolega PhD^1,6^, James N. Jarvis MD^3,7^, Hui Meng PhD^1,2,4,8^, *Vincent M. Tutino PhD^1,4,6^

**Affiliations**: ^1^Canon Stroke and Vascular Research Center; Jacobs School of Medicine & Biomedical Sciences, University at Buffalo, Buffalo, New York, USA

^2^Department of Biomedical Engineering, University at Buffalo, Buffalo, New York, USA

^3^Genetics, Genomics, and Bioinformatics Program, Jacobs School of Medicine & Biomedical Sciences, University at Buffalo, Buffalo, New York, USA

^4^Department of Neurosurgery, Jacobs School of Medicine & Biomedical Sciences, University at Buffalo, Buffalo, New York, USA

^5^Department of Radiology, Jacobs School of Medicine & Biomedical Sciences, University at Buffalo, Buffalo, New York, USA

^6^Department of Pathology and Anatomical Sciences, Jacobs School of Medicine & Biomedical Sciences, University at Buffalo, Buffalo, New York, USA

^7^Department of Pediatrics, Jacobs School of Medicine & Biomedical Sciences, University at Buffalo, Buffalo, New York, USA

^8^Department of Mechanical & Aerospace Engineering, University at Buffalo, Buffalo, New York, USA

***Correspondence**

Vincent M. Tutino, PhD

Clinical and Translational Research Center, CSRVC

875 Ellicott Street

Buffalo, NY 14214 USA

E-mail:[vincentt@buffalo.edu](mailto:vincentt@buffalo.edu); Phone:(716) 829-5400; Fax:(716) 854-1850

**Table S1: Entire list of significant ontologies from GO::TermFinder***

|  | **Term** | **Q-value** | **FDR** | **Genes** |
| --- | --- | --- | --- | --- |
| ***Gene Ontology Process*** | |  |  |  |
|  | Regulation of endopeptidase activity | 0.0048 | 0.00% | *SERPINA3, SERPINA12, SERPINA9, SERPINA11, HSPD1, SERPINA4, HSPE1, SERPINA13P,  SERPINA5* |
|  | Regulation of peptidase activity | 0.0081 | 4.67% | *SERPINA3, SERPINA12, SERPINA9, SERPINA11, HSPD1, SERPINA4, HSPE1, SERPINA13P,  SERPINA5* |
| ***Gene Ontology Function*** | |  |  |  |
|  | Serine-type endopeptidase inhibitor activity | 4.04E-06 | 0.00% | *SERPINA4, SERPINA3, SERPINA12, SERPINA9, SERPINA13P, SERPINA11, SERPINA5* |
|  | Endopeptidase inhibitor activity | 0.00025 | 0.00% | *SERPINA4, SERPINA3, SERPINA12, SERPINA9, SERPINA13P, SERPINA11, SERPINA5* |
|  | Endopeptidase regulator activity | 0.00031 | 0.00% | *SERPINA4, SERPINA3, SERPINA12, SERPINA9, SERPINA13P, SERPINA11, SERPINA5* |
|  | Peptidase inhibitor activity | 0.00034 | 0.00% | *SERPINA4, SERPINA3, SERPINA12, SERPINA9, SERPINA13P, SERPINA11, SERPINA5* |
|  | Extracellular matrix structural constituent | 0.00065 | 0.00% | *COL1A2, HAPLN1, VCAN, COL5A2, COL3A1* |
|  | Peptidase regulator activity | 0.0011 | 0.00% | *SERPINA4, SERPINA3, SERPINA12, SERPINA9, SERPINA13P, SERPINA11, SERPINA5* |
|  | Enzyme inhibitor activity | 0.0065 | 0.25% | *SERPINA3, SERPINA12, SERPINA9, SERPINA11, CDKN2B, SERPINA4, SERPINA13P, SERPINA5* |
| ***Gene Ontology Component*** | |  |  |  |
|  | Fibrillar collagen trimer | 0.0010 | 0.00% | *COL1A2, COL5A2, COL3A1* |
|  | Banded collagen fibril | 0.0010 | 0.00% | *COL1A2, COL5A2, COL3A1* |
|  | Complex of collagen trimers | 0.0053 | 0.00% | *COL1A2, COL5A2, COL3A1* |

***GO::TermFinder results for molecular function, biological process, and cellular component ontologies using genes within ±200kb of intracranial aneurysm-associated linkage disequilibrium blocks. Unannotated terms have been removed.

Abbreviations: FDR=false discovery rate, GO::TermFinder=Gene Ontology Term Finder

**Figure S1**

*

*

**Figure S1: Gene Ontology term finder molecular process results for genes within extended intracranial aneurysm-associated linkage disequilibrium blocks.** Blocks in blue represent significant ontologies.

**Figure S2**

*
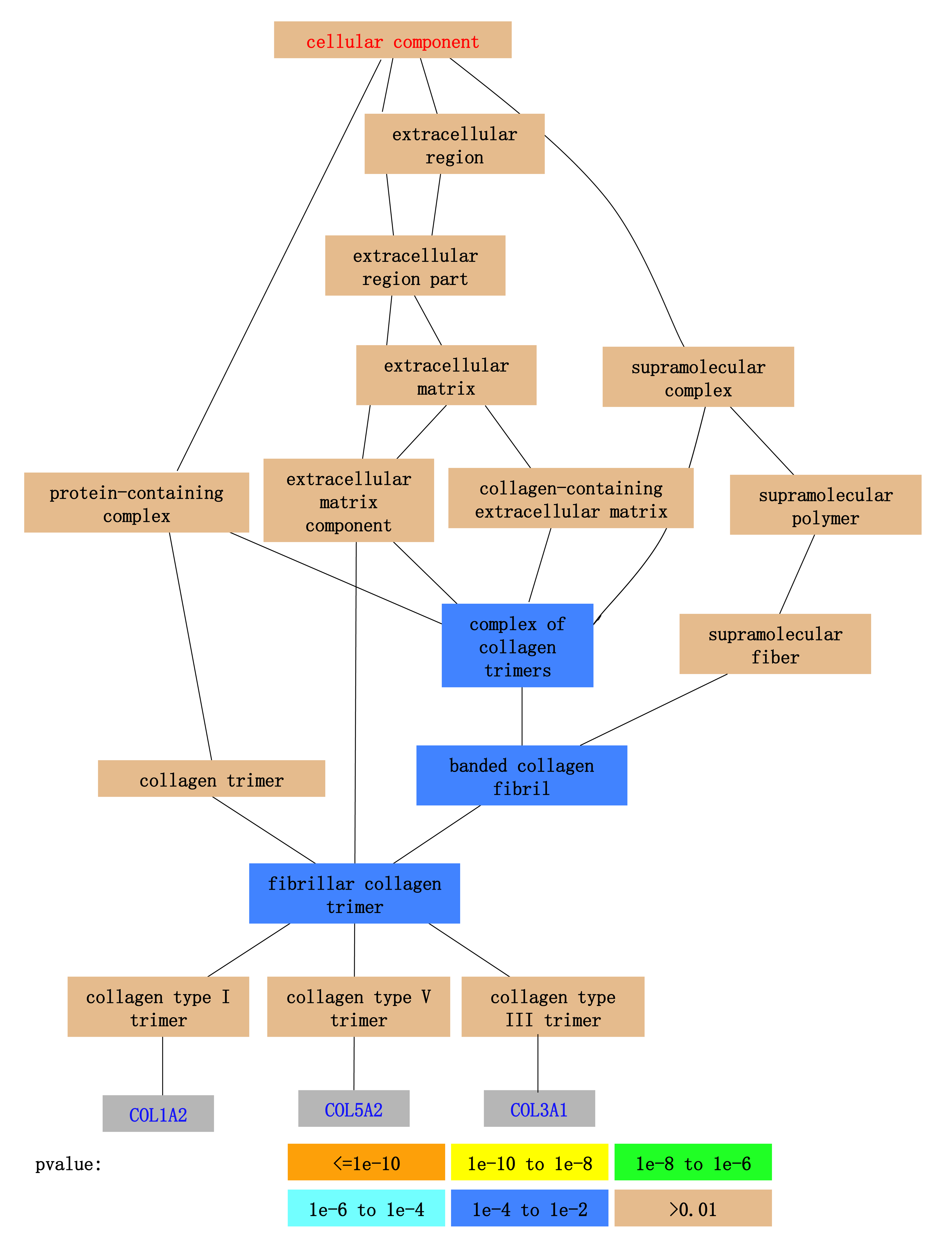
*

**Figure S2: Gene Ontology term finder molecular component results for genes within extended intracranial aneurysm-associated linkage disequilibrium blocks.** Blocks in blue represent significant ontologies.
